# Key Role of the Membrane Trafficking of Nav1.5 Channel Protein in Antidepressant-Induced Brugada Syndrome

**DOI:** 10.3389/fphys.2018.01230

**Published:** 2018-09-05

**Authors:** Xi Chen, Chao Zhu, Hao Zhou, Yu Zhang, Zhongqi Cai, Honglin Wu, Xiaomeng Ren, Lei Gao, Jiancheng Zhang, Yang Li

**Affiliations:** ^1^Department of Geriatrics, The First Affiliated Hospital of Chongqing Medical University, Chongqing, China; ^2^Department of Cardiology, Chinese People’s Liberation Army General Hospital, Beijing, China; ^3^Department of Cardiology, Provincial Clinical Medicine College of Fujian Medical University, Fuzhou, China

**Keywords:** Brugada syndrome, antidepressant, Nav1.5, long-term effect, trafficking, interacting proteins

## Abstract

Anti-depressant treatment has been found to be associated with the development of Brugada syndrome (BrS) through poorly defined mechanisms. Herein, this study aimed to explore the molecular basis for amitriptyline-induced BrS. The effects of long-term treatments of amitriptyline on Nav1.5 were investigated using neonatal rat ventricular myocytes. The electrophysiological properties, expression and distribution of Nav1.5 were studied using the patch clamp, Western blot and confocal laser microscopy assays. Interactions between Nav1.5 and its interacting proteins, including ankyrin-G and dystrophin, were evaluated by co-immunoprecipitation. A larger decrease in the peak I_Na_ occurred after long-term treatments to amitriptyline (56.64%) than after acute exposure to amitriptyline (28%). Slow recovery from inactivation of Nav1.5 was observed after acute or long-term treatments to amitriptyline. The expression of Nav1.5 on the cell membrane showed a larger decrease by long-term treatments to amitriptyline than by acute exposure to amitriptyline. After long-term treatments to amitriptyline, we observed reduced Nav1.5 proteins on the cell membrane and the disrupted co-localization of Nav1.5 and ankyrin-G or dystrophin. Co-immunoprecipitation experiments further testified that the combination of Nav1.5 and ankyrin-G or dystrophin was severely weakened after long-term treatments to amitriptyline, implying the failed interaction between Nav1.5 and ankyrin-G or dystrophin. Our data suggest that the long-term effect of amitriptyline serves as an important contribution to BrS induced by amitriptyline. The mechanisms of BrS induced by amitriptyline were related to Nav1.5 trafficking and could be explained by the disrupted interaction of ankyrin-G, dystrophin and Nav1.5.

## Introduction

Brugada syndrome (BrS) is an inherited arrhythmia disease diagnosed by the ECG findings of ST-segment elevation in the right precordial lead, without identifiable structural abnormalities (Priori et al., 2013). The prevalence of BrS in Southeast Asians is estimated to be 0.1%, much higher than in other ethnicities (Ng et al., 2012). In the Chinese population, its prevalence was demonstrated to be 3.3% in a recent epidemiological study (Juang et al., 2015). Despite the low prevalence, BrS is strongly associated with the increased risk of ventricular fibrillation and sudden cardiac death (SCD) (Murakoshi and Aonuma, 2013).

Intensive research has emphasized the concern that antidepressants increase the risk of BrS. Overall, more than 16 antidepressants have been identified to be associated with BrS and related SCD^1^. Among the cases of cyclic antidepressant application, the prevalence of a Brugada electrocardiographic pattern was reported to be 15.3% (Goldgran-Toledano et al., 2002). It should be noted that amitriptyline and nortriptyline use was associated with 2.5-fold and 4.5-fold increases, respectively, in the risk of SCD (Ray et al., 2004; Bardai et al., 2013). These facts underlie the importance of revealing the mechanisms of antidepressant-induced BrS that remain poorly understood.

Previous studies on antidepressant-induced BrS mainly focused on the altered gating properties of the alpha subunit of the voltage-gated Nav1.5 cardiac sodium channel, such as a decreased peak current and delayed recovery from inactivation (Minoura et al., 2012). However, the Brugada ECG pattern or symptomatic BrS in antidepressant users often occurred after the long-term use of these drugs (Chow et al., 2005; Kofune et al., 2013). Thus, the gating property changes of Nav1.5 may not well explain these clinical manifestations. Since a recent study discovered a 15-fold increase in I_Na-L_ by chronic exposure to dofetilide but not by acute exposure to dofetilide (Yang et al., 2014), increasing attention has been given to the long-term treatments effect of drugs. Therefore, we speculate that apart from the acute effect, the long-term treatments of antidepressants may better explain the induction of BrS in antidepressant users.

To fulfill the physiological function, the Nav1.5 channel should be delivered from the endoplasmic reticulum to the Golgi apparatus and finally to specific subdomains of the plasma membrane, namely, the process of trafficking (Herfst et al., 2004). Sodium channel-interacting proteins such as ankyrin-G and dystrophin were proven to be essential to maintain the precise process of Nav1.5 trafficking. Trafficking of Nav1.5 to the cell membrane could be disrupted in ankyrin-G-knockout myocytes, and ventricular arrhythmia was observed in ankyrin-G-knockout mice (Makara et al., 2014). Deficiency of dystrophin could account for an almost 30% decrease in the sodium current (Gavillet et al., 2006). Thus, this study tests the hypothesis that antidepressants may have a long-term blockade effect on Nav1.5, which may be due to the fault of Nav1.5 trafficking to the cell membrane, with the regulation of the sodium channel-interacting proteins ankyrin-G and dystrophin.

## Materials and Methods

### Neonatal Rat Cardiomyocyte Isolation

Twenty-four-hour-old neonatal Sprague–Dawley rats were purchased from the animal center of SiBeiFu in Beijing, China. The protocol of the study was approved by the animal ethics committee of the Chinese People’s Liberation Army General Hospital in accordance with NIH guidelines. We used a modified method according to previous reports (Kadota et al., 2017; Wu et al., 2017). Isolated ventricles of the hearts from the rats were placed in cold phosphate-buffered saline (PBS) (HyClone, United States). Next, the ventricles were minced and digested in a protease solution (0.025% type 2 collagenase/0.08% pancreatin, Gibco, United States) at 37°C. Fetal bovine serum (FBS) (Gibco, United States) was used to terminate the digestion. Digestion was repeated 8–10 times, for 2∼5 min each. The cells in the supernatant were collected by centrifugation at 1500 rpm for 10 min. The collected cells were then cultured in Dulbecco’s modified Eagle’s medium (DMEM) (HyClone, United States) supplemented with 5% FBS (Gibco, United States) and 1% streptomycin (100 mg/ml)/penicillin (100 U/ml) (Gibco, United States). After incubation for 2 h, ventricular myocytes were separated from cardiac fibroblasts (CFs). Cells in the supernatant were subsequently plated in cell culture dishes at 37°C with 5% CO_2_. The culture medium was supplemented with 30 μg/ml of bromodeoxyuridine (Sigma, United States) to prevent CF growth.

For the acute effect of the drugs, the cells were directly superfused with drugs for 5 min, and the I_Na_ of the cells was recorded. For the long-term effect of the drugs, with coincubation of the drugs for 24 h, the I_Na_ of the cells was recorded. The drugs used were amitriptyline (1.0 μM), clomipramine (1.0 μM), nortriptyline (1.0 μM), and desipramine (1.0 μM) (Sigma, United States).

### Patch Clamp

All whole-cell recordings were obtained at room temperature, utilizing an Axon Multiclamp 700B Amplifier (Molecular Devices, United States). Signal acquisition was completed using a digidata 1440A acquisition interface (Molecular Devices, United States) controlled by pCLAMP programs (version 10.2). Isolated neonatal rat ventricular myocytes were incubated for 24 h before the incubation solution was replaced by an extracellular solution for the patch experiment. The extracellular solution contained the following: 116 mM of NaCl, 20 mM of TEA-Cl, 3 mM of KCl, 1 mM of CaCl_2_, 1 mM of MgCl_2_.6H_2_O, 10 mM of HEPES, 10 mM of glucose, 0.1 mM of CdCl_2_, and 4 mM of choline chloride, with the pH adjusted to 7.3 with NaOH. The pipette solution contained the following: 5 mM of NaCl, 135 mM of CsCl, 10 mM of EGTA, 5 mM of Na_2_ATP, 5 mM of MgCl_2_.6H_2_O, and 5 mM of HEPES, with the pH adjusted to 7.2 with NaOH. The pipette resistance ranged from 2 to 3 MΩ and was maintained to record I_Na_. The series resistance was compensated by 90∼95%, and slow capacitance was compensated by 85∼90% to minimize the voltage clamp errors.

To determine the voltage dependence of the peak I_Na_, cells were held at -120 mV, and 300-ms voltage steps were applied from -90 to +40 mV by 10-mV increments. The peak I_Na_ was then divided by the membrane capacitance to analyze the peak current density. To determine the voltage dependence of activation, normalized I_Na_ was plotted as voltage-dependent activation curves fitted to the Boltzmann distribution. To determine the voltage-dependent inactivation, I_Na_ was elicited by application of 1000-ms conditioning pulses from -150 mV to 0 mV, followed by a 30-ms test pulse to -35 mV. Voltage-dependent inactivation curves were fitted to the Boltzmann distribution. To determine the recovery from inactivation, paired test pulses to -35 mV were applied, each for 30 ms, with increasing intervals of 40 ms to a maximum of 740 ms between the paired pulses and a holding potential of -120 mV. The time-dependent recovery curves were fitted to exponential functions. For analysis of the intermediate-state inactivation, pre-pulses to -20 mV with increasing intervals ranging from 1 to 3200 ms were applied, followed by a 50-ms test pulse to -20 mV. For analysis of the closed-state inactivation, pre-pulses to -100 mV with increasing intervals ranging from 1 to 500 ms were applied, followed by a 50-ms pulse to -20 mV.

### Confocal Imaging

As described previously (Casini et al., 2010), cells were rinsed with cold PBS twice and then were fixed with 4% polyformaldehyde for 15 min at 4°C. After rinsing with cold PBS again, the cells were incubated in PBS solution containing 10% normal goat serum, 1% bovine serum albumin (BSA) and 0.1% TritonX-100 to block non-specific sites. Primary antibodies or secondary antibodies were diluted in a PBS solution containing 3% normal goat serum and 1% BSA. Cells were incubated with primary antibodies at 4°C overnight and then were incubated with secondary antibody at room temperature for 2 h. The antibodies used in the present study were as follows: mouse-anti-ankyrin-G (Santa Cruz, United States), mouse-anti-dystrophin (Sigma, United States), rabbit-anti-Nav1.5 (Alomone Labs), goat-anti-rabbit RBITC and goat-anti-mouse FITC. Finally, the samples were examined using a Leica TCS-SP2 digital scanning confocal microscope.

### Western Blotting

This method was described previously (Zhang et al., 2015; Chen et al., 2016). Briefly, the collected neonatal ventricular myocytes were rinsed with cold PBS and were lysed in RIPA (Radio Immuno Precipitation Assay) buffer (Sigma, United States) containing proteinase inhibitor to prepare the cell lysates. The lysates were then centrifuged at 700 *g* at 4°C for 10 min. The supernatants were collected for an additional centrifugation (10,000 *g*, 4°C for 10 min). The plasma membrane protein extraction kit (Biovision, United States) was used for total membrane protein extraction. The protein concentration was measured using the BCA (bicinchoninic acid) protein assay kit (Sigma, United States). The proteins were separated by electrophoresis on SDS-polyacrylamide gels, transferred to nitrocellulose membranes and incubated with primary antibodies against Nav1.5, ankyrin-G and dystrophin at 4°C overnight. After rinsing, the blots were incubated with the appropriate horseradish peroxidase-conjugated secondary antibody at 24°C for 1 h. Chemiluminescence detection was performed with substrate reagents by a CCD camera. Densitometric analysis was performed with Bandscan 5.0 software. For quantification, the protein expression levels were normalized to the GAPDH levels and against controls. Each experiment was repeated at least three times.

### Co-immunoprecipitation

Cells were lysed in RIPA buffer (Sigma, United States) supplemented with proteinase inhibitor. After rotating at 4°C for 30 min, the homogenates were centrifuged at 16000 rpm at 4°C for 20 min. The supernatants were incubated with the primary antibody at 4°C for 2 h. Next, the immune complexes were incubated with protein A/G beads at 4°C overnight. After centrifugation, the supernatants were discarded, and the immunoprecipitates were rinsed for Western blotting. Each experiment was repeated at least three times.

### Statistical Analysis

Off-line leak correction was performed on all of the amplitude data. The data are presented as mean values ± SD., with *n* representing the number of cells analyzed. pCLAMP version 10.2 (Axon Instruments) and Origin (Microcal Software) were used for the data analysis. *P*-values < 0.05 were considered significant, and statistical analyses between the experimental groups were performed using Student’s *t*-test. One-way analysis of variance (ANOVA) was used when comparing multiple groups, and the significance between any two groups was evaluated by ANOVA followed by the Student–Newman–Keuls (S–N–K) *post hoc* test. The SPSS computer program (version 17.0) was used for the analyses. SSA curves were fitted using a Boltzmann distribution as follows: *G*_(t)_/*G*_max_ = 1/(1 × Exp[(*V*_m_-*V*_1/2_)/*k*]. The conductance value was computed using the following equation: *G* = *I*_max_/(*V*_m_-*E*_Na_), where *G* is the conductance, *G*_max_ is the maximum conductance value, *I*_max_ represents the peak test pulse current, *V*_m_ represents the test pulse voltage, and *E*_Na_ represents the measured equilibrium potential for sodium. The SSIs were fitted using the Boltzmann equation, *I*_(t)_/*I*_max_ = [1 × Exp (-(*V*_m_-*V*_1/2_)/*k*)], to determine the membrane potential for half-maximal inactivation (*V*_1/2_) and the slope factor *k*. *I*_(t)_ represents the test pulse potentials, *I*_max_ represents the peak test pulse current, and *V*_m_ is the test pulse/pre-pulse potential. Recovery from inactivation was assessed using the double-pulse protocol shown in the inset and fitted using a single-exponential function *I*_(Δt)_/*I*_max_ = AExp(Δ*t*/*τ*), where the values for A refer to the amplitudes, and those for *τ* refer to the time constants. *I* refers to the current, and *t* refers to time. All the data were fitted using a nonlinear least-squares minimization method.

## Results

### Acute and Long-Term Effects of Amitriptyline on *I*_Na_

**FIGURE 1:**
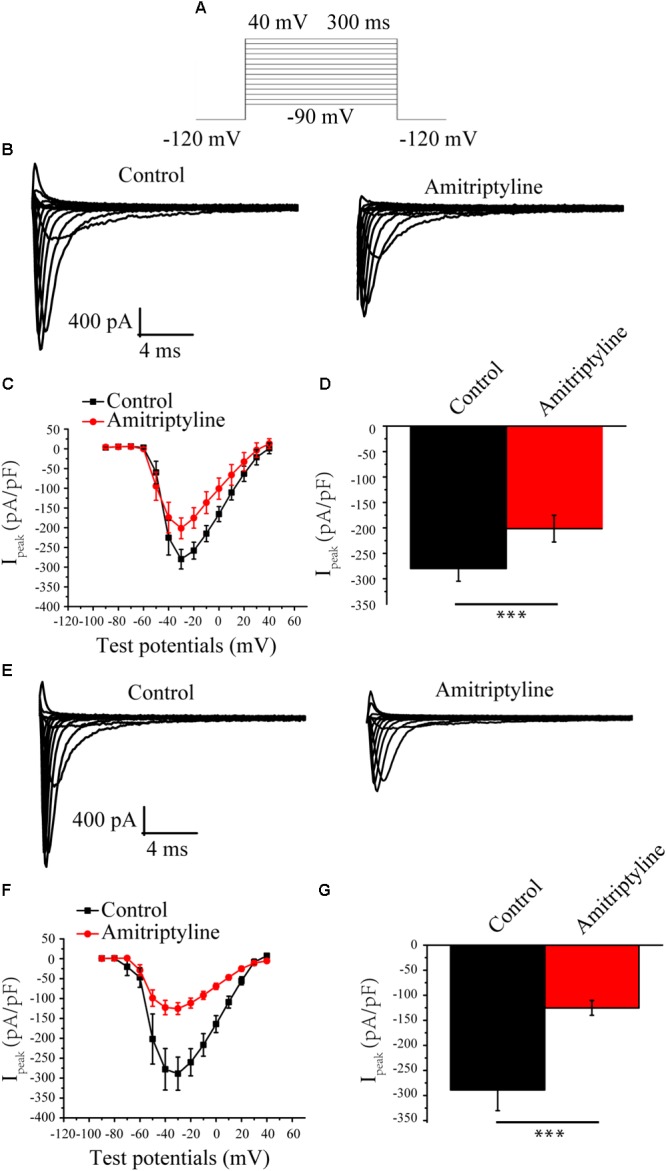
Effect of amitriptyline on Nav1.5 currents. **(A)** Protocols for I_Na_ eliciting. **(B)** Representative I_Na_ in control cells and in cells with acute treatments of amitriptyline. **(C)** *I-V* curves for control cells and cells with acute treatments of amitriptyline. **(D)** Analysis of peak I_Na_ densities for the acute effect of amitriptyline. **(E)** Representative I_Na_ in control cells and cells with long-term treatments of amitriptyline. **(F)** *I-V* curves for control cells and cells with long-term treatments of amitriptyline. **(G)** Analysis of peak I_Na_ densities for the long-term effect of amitriptyline. ^∗∗∗^*P* < 0.001.

*I*_Na_ was elicited by the protocols shown in **Figure 1A**. Representative *I*_Na_ before and after treatments with amitriptyline are shown in **Figure 1B** (acute effect) and **Figure 1E** (long-term effect). After acute treatments with amitriptyline, the *I*_Na_ densities were decreased, and at -35 mV of the test potential, the peak *I*_Na_ densities were reduced from -279.9 ± 24.9 pA/pF to -201.5 ± 26.3 pA/pF (*P* < 0.001, *n* = 6) (**Figures 1C,D**). With coincubation with amitriptyline for 24 h, the *I*_Na_ densities were decreased. At -35 mV of the test potential, the peak *I*_Na_ densities were decreased from -288.8 ± 41.5 pA/pF to -125.2 ± 14.8 pA/pF (*P* < 0.001, *n* = 15, **Figures 1F,G**). An obvious decrease in *I*_Na_ induced by amitriptyline (both acute and long-term treatments) was observed at test potentials ranging from -40 mV to -10 mV. It is interesting that the reduction of *I*_Na_ with the coincubation of amitriptyline for 24 h was twice that of the directly superfused with drug for 5 min. At the test potential of -35 mV, the peak *I*_Na_ was decreased by 28.2 and 56.6% due to acute and long-term treatments with amitriptyline, respectively (**Figures 1D,F**). The results suggested a stronger effect on *I*_Na_ induced by the long-term effect of amitriptyline.

### Effect of Amitriptyline on the Gating Properties of *I*_Na_

**FIGURE 2:**
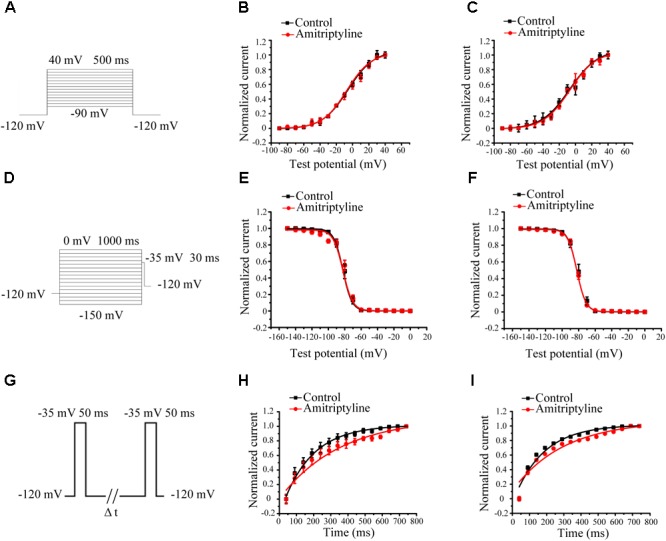
Effect of amitriptyline on the gating properties of Nav1.5 currents. **(A)** Protocols for steady-state activation (SSA) recording. **(B)** SSA curves for the acute effect of amitriptyline. **(C)** SSA cures for the long-term effect of amitriptyline. **(D)** Protocols for steady-state inactivation (SSI) recording. **(E)** SSI curves for the acute effect of amitriptyline. **(F)** SSI curves for the long-term effect of amitriptyline. **(G)** Protocols for recovery from inactivation (RFI) recording. **(H)** RFI curves for the acute effect of amitriptyline. **(I)** RFI curves for the long-term effect of amitriptyline.

Steady-state activation (SSA) of *I*_Na_ was evaluated by a protocol shown in **Figure 2A**. After acute treatments with amitriptyline, *V*_1/2_ did not shift significantly, and *k* did not change significantly. After long-term treatments with amitriptyline, there was no significant shift of *V*_1/2_ and *k* (**Figures 2B,C**).

Steady-state inactivation (SSI) was evaluated by a protocol shown in **Figure 2D**. The SSI processes of *I*_Na_ was not significantly changed with acute and long-term treatments of amitriptyline. Compared with controls, *V*_1/2_ or *k* were not markedly different either directly superfused or with coincubation for 24 h with amitriptyline (**Figures 2E,F**).

Recovery from inactivation (RFI) was evaluated by a protocol shown in **Figure 2G**. The recovery time constants from the inactivation of *I*_Na_ were affected by amitriptyline. The acute effect of amitriptyline caused significant prolongation of the recovery time constants (from 120.8 ± 6.2 ms to 244.7 ± 16.1 ms, *P* < 0.01, *n* = 6). Similarly, the long-term effect of amitriptyline also showed a prolongation of the recovery time constant from 120.7 ± 9.2 ms to 206.9 ± 11.2 ms (*P* < 0.01, *n* = 15, **Figures 2H,I**).

### Effect of Amitriptyline on Intermediate-State Inactivation and Closed-State Inactivation of *I*_Na_

**FIGURE 3:**
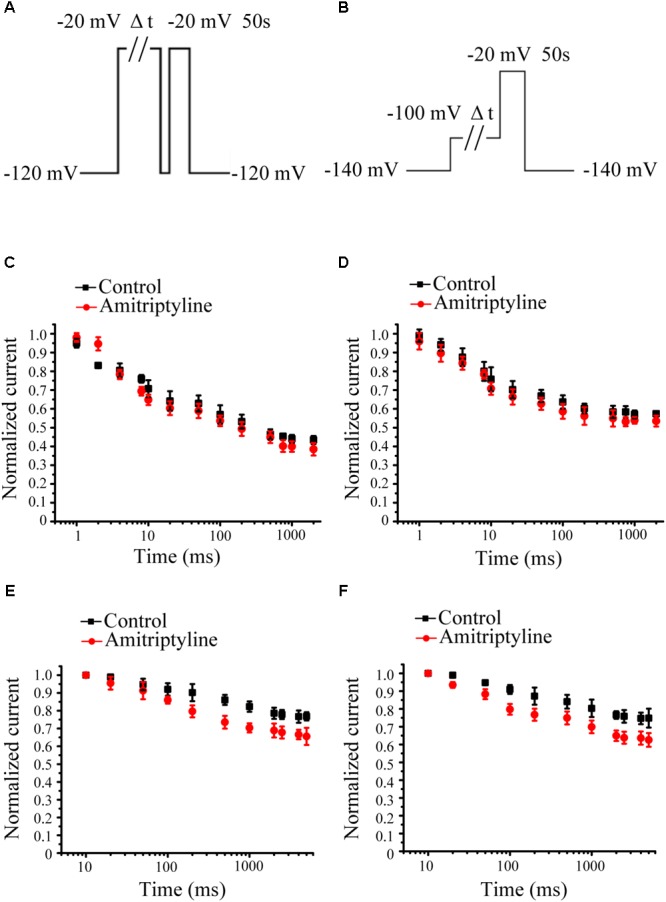
Effect of amitriptyline on intermediate-state inactivation (ISI) and closed-state inactivation (CSI) of Nav1.5 currents. **(A)** Protocols for ISI recording. **(B)** Protocols for CSI recording. **(C)** ISI curves for the acute effect of amitriptyline. **(D)** ISI cures for the long-term effect of amitriptyline. **(E)** CSI curves for the acute effect of amitriptyline. **(F)** CSI cures for the long-term effect of amitriptyline.

Intermediate-state inactivation and closed-state inactivation were evaluated by protocols shown in **Figures 3A,B**. Neither the acute effect of amitriptyline nor the long-term effect of amitriptyline had an obvious impact on the intermediate-state inactivation of Nav1.5 currents (**Figures 3C,D**). However, the acute effect of amitriptyline and long-term effect of amitriptyline prolonged the time constants of the closed-state inactivation to a similar extent (**Figures 3E,F**).

### Cellular Membrane Expression and Distribution of the Nav1.5 Channel Protein After Long-Term Treatments With Amitriptyline

**FIGURE 4:**
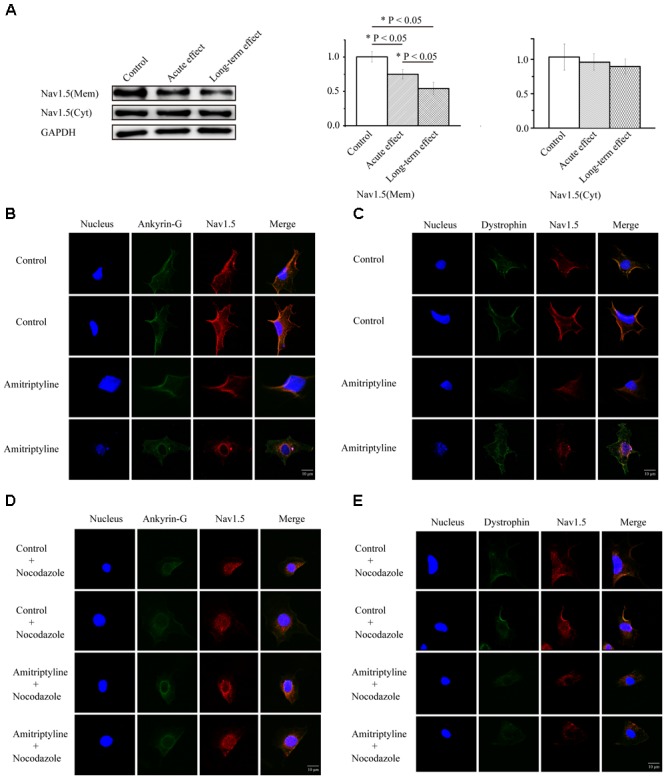
Long-term effect of amitriptyline on protein expression and distribution of Nav1.5. **(A)** Protein expression of Nav1.5 in control cells, cells with acute treatments of amitriptyline and cells with long-term treatments with amitriptyline. **(B)** Representative confocal images of Nav1.5 (red) and ankyrin-G (green) without or with long-term treatments with amitriptyline. **(C)** Representative confocal images of Nav1.5 (red) and dystrophin (green) without or with long-term treatments with amitriptyline. **(D)** Representative confocal images of Nav1.5 and ankyrin-G with nocodazole treatments. **(E)** Representative confocal images of Nav1.5 and dystrophin with nocodazole treatments. The blue signal indicates the nucleus. Scale bar: 10 μm. ^∗^*P* < 0.05.

Compared with the controls, both the acute effect of amitriptyline and long-term effect of amitriptyline caused significantly less Nav1.5 protein expressed on the cell membrane (1.00 ± 0.07 vs. 0.75 ± 0.07 vs. 0.54 ± 0.09, *P* < 0.05, *n* = 3). However, the Nav1.5 protein on the cell membrane was decreased more after long-term treatments with amitriptyline (0.54 ± 0.09) than after acute treatments with amitriptyline (0.75 ± 0.07, *P* < 0.05, *n* = 3) (**Figure 4A**). We did not observe significant reduction of cytosolic Nav1.5 protein compared with controls, after either acute or long-term treatments with amitriptyline (1.03 ± 0.07 vs. 0.96 ± 0.05 vs. 0.90 ± 0.05, *P* > 0.05, *n* = 3). Immunostaining experiments showed that the red signal intensity, which represented Nav1.5 protein, was distributed both in the cytoplasm and on the cell membrane. Nav1.5 on the cell membrane was observed clearly before long-term treatments with amitriptyline and was reduced markedly after long-term treatments with amitriptyline. The results suggested that the long-term effect of amitriptyline caused the retention of Nav1.5 protein within the cell.

In addition, in cells without treatments of amitriptyline, we observed that Nav1.5 protein was localized together with ankyrin-G (**Figure 4B**) and dystrophin (**Figure 4C**) on the cell membrane. However, after long-term treatments with amitriptyline, the co-localization of Nav1.5 with ankyrin-G or dystrophin could not be clearly observed. To further investigate whether trafficking dysfunction was related to the long-term effect of amitriptyline, we pre-treated all cells with nocodazole. Next, we observed similar a distribution of Nav1.5, ankyrin-G and dystrophin between the groups with or without long-term treatments with amitriptyline (**Figures 4D,E**).

### Disrupted Interaction of Ankyrin-G, Dystrophin and Nav1.5 After Long-Term Treatments With Amitriptyline

**FIGURE 5:**
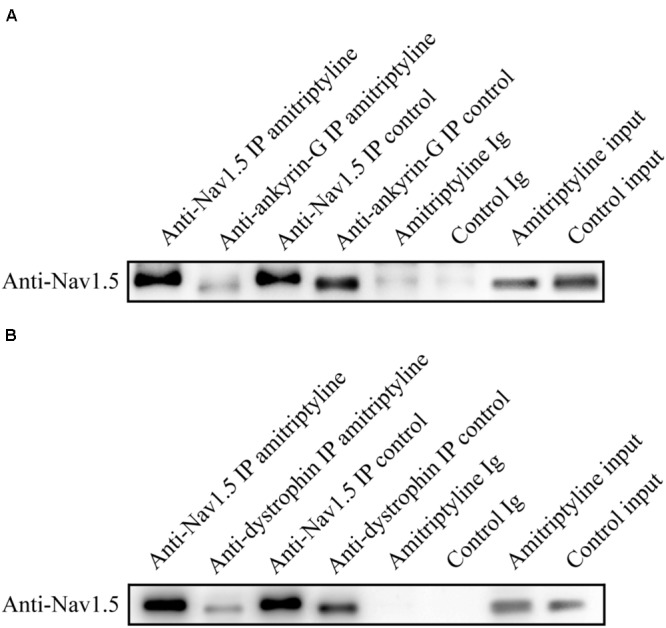
Long-term effect of amitriptyline on the Nav1.5-ankyrin-G interaction and Nav1.5-dystrophin interaction. **(A)** With the anti-ankyrin-G antibody, Nav1.5 was detectable without long-term treatments with amitriptyline but was not clearly observed after long-term treatments with amitriptyline. **(B)** With anti-dystrophin antibody, Nav1.5 was detectable without long-term treatments with amitriptyline but was not clearly observed after the long-term treatments with amitriptyline. Input: Western blotting of total protein lysates.

Co-immunoprecipitation experiments were used to test the Nav1.5-ankyrin-G interaction and Nav1.5-dystrophin interaction. Anti-Nav1.5 antibody was used to analyze the immuno-complex. Nav1.5 protein could be precipitated by anti-ankyrin-G antibody and anti-dystrophin antibody before exposure to amitriptyline. However, the Nav1.5 protein bands could hardly be observed in the immuno-complex precipitated either by the anti-ankyrin-G antibody (**Figure 5A**) or anti-dystrophin antibody (**Figure 5B**) after 24 h of incubation with amitriptyline. These results suggested that the interaction between Nav1.5 and ankyrin-G or dystrophin could be weakened by the long-term effect of amitriptyline.

### Effect of Several Other Antidepressants on *I*_Na_

**FIGURE 6:**
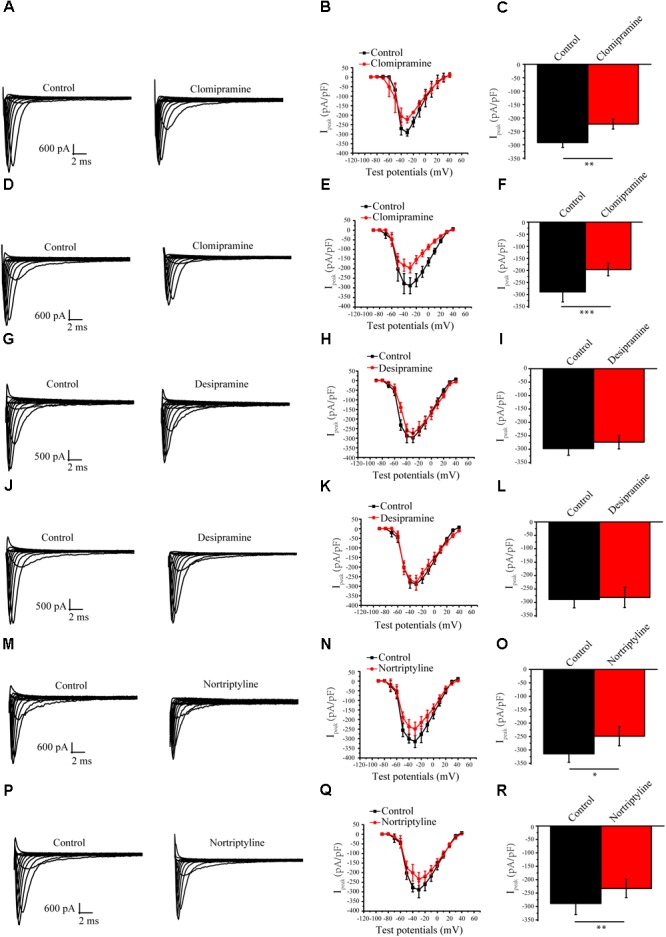
Effect of other antidepressants on Nav1.5 currents. **(A)** Representative I_Na_ current traces recorded from cells under control conditions and acute clomipramine treatments of **(B,C)** *I-V* curves and analysis of the peak I_Na_ densities for the acute effect of clomipramine. **(D)** Representative I_Na_ current traces recorded from cells with long-term treatments of clomipramine. **(E,F)** *I-V* curves and peak I_Na_ densities of cells with long-term treatmentsn of clomipramine. **(G–I)** Representative I_Na_ in cells with acute desipramine treatments. **(J–L)** Representative I_Na_ in cells with long-term treatments of desipramine. **(M–R)** Representative I_Na_ in cells with acute and long-term treatments of nortriptyline. ^∗^*P* < 0.05, ^∗∗^*P* < 0.01.

To determine whether the above long-term effects are specific to amitriptyline, we examined the effects of several other antidepressants on the sodium current. The results showed that clomipramine showed similar response characteristics as amitriptyline regarding *I*_Na_. A larger decline in the peak *I*_Na_ was shown to be induced more by long-term treatments with clomipramine (32.0%) than by acute treatments (23.6%). However, the results observed with the other two antidepressants were not always consistent with those with amitriptyline. The acute effect of nortriptyline caused a 20.9% decrease in *I*_Na_, and the long-term effect caused a 19.3% decrease. *I*_Na_ was not significantly changed by both acute or long-term treatments with desipramine (**Figure 6**).

## Discussion

Amitriptyline has been widely used and associated with BrS, whereas the mechanisms remain poorly understood. To the best of our knowledge, the present study confirmed for the first time that the long-term effect might be an important contribution to BrS induced by amitriptyline. The underlying mechanisms involved the impairment of Nav1.5 trafficking, which could be regulated by the Nav1.5-ankyrin-G interaction and Nav1.5-dystrophin interaction.

The concentrations of antidepressants used in this study are near therapeutic plasma concentration used in the clinic. Actually, in patients receiving daily doses of 75–300 mg of amitriptyline, plasma steady-state concentrations range from 0.3 to 0.9 μM (Pancrazio et al., 1998). The concentration of 1 μM nortriptyline exposed to ventricular myocytes is equivalent to serum levels in patients during chronic use of 150 mg nortriptyline (Bardai et al., 2013). In the present study, we used the same concentration of amitriptyline as previous studies to explore the electrophysiological and molecular biological changes of cardiac sodium cannels induced by amitriptyline (Nau et al., 2000).

The present study demonstrated that the long-term blockade effect of amitriptyline on Nav1.5 was stronger than the acute blockade effect. Similar to our previous study, the rescue effect of alpha-allocryptopine on the SCN5A-T353I current was observed only by coincubation with alpha-allocryptopine for 24 h but not by direct perfusion of alpha-allocryptopine (Zhang et al., 2015). There were also other studies illustrating similar results for the long-term effects of drugs. Dofetilide showed a strong capacity to increase the late sodium current only through exposure for 48 h (Chen et al., 2016). Desipramine imposed an acute effect, a short-term effect or a long-term effect according to the different exposure times on hERG channels (Staudacher et al., 2011). Thus, a stronger long-term effect other than the acute effect of amitriptyline turned out to be an important contribution to the occurrence of BrS and a better explanation for the clinical manifestations.

Consistent with previous reports (Wang et al., 2004; Donovan et al., 2011; Liang et al., 2014), the slow recovery from the inactivated state was the main gating property alteration demonstrated in the present study. The cause might involve the high affinity of amitriptyline for inactivated-state sodium channels (Nau et al., 2000). However, no shift of the SSI curves induced by amitriptyline was detected in the present study, a finding that was in contrast to the previous findings that SSI curves were shifted to the negative direction by the application of amitriptyline (Minoura et al., 2012). Such inconsistency may result from different protocols and cell sources. The neonatal rat ventricular myocytes used in this study were expected to share more electrophysiological characteristics with human myocytes than cells transfected with cardiac sodium channels. Despite the similar changes in the gating properties imposed by acute and long-term effects of amitriptyline, the long-term effect of amitriptyline caused a much larger decline in the peak sodium current. Thus, the altered gating properties may not fully explain this phenomenon, indicating other underlying mechanisms.

In the present study, we found a decreased expression and distribution of Nav1.5 protein on the cell membrane, as well as the retention of Nav1.5 protein within cells after long-term treatments with amitriptyline. With the application of nocodazole, an inhibitor for channel trafficking (Zhang et al., 2014), the impact of amitriptyline was almost covered. Such a close relationship between long-term drug incubation and its effect on channel trafficking implied in this study has been revealed previously. As stated previously, desipramine could exert different impacts on hERG channels such as gating property changes and trafficking dysfunction, depending on the exposure time to the drug (Staudacher et al., 2011). Our previous study also found that after coincubation with cells for 24 h, alpha-allocryptopine had the capacity to rescue trafficking dysfunction of SCN5A-T353I (Zhang et al., 2015). As deduced from these findings, the novel mechanism responsible for the long-term blockade effect of amitriptyline on Nav1.5 was the impairment of Nav1.5 trafficking.

Furthermore, we observed less co-localization and weaker binding between Nav1.5 and ankyrin-G or dystrophin after the long-term treatments with amitriptyline, indicating the disrupted Nav1.5-ankyrin-G interaction and Nav1.5-dystrophin interaction induced by amitriptyline. Actually, a conserved ankyrin-binding motif was detected in Nav1.5 protein (Garrido et al., 2003; Lemaillet et al., 2003). The loss of ankyrin-G could cause a decrease in the sodium current and Nav1.5 surface expression without affecting channel gating properties, owing to the requirements of ankyrin-G for Nav1.5 trafficking (Lowe et al., 2008). Interestingly, the binding site between Nav1.5 and ankyrin-G was confirmed to be somewhere near E1053 (Mohler et al., 2004), while the binding site between Nav1.5 and amitriptyline was proven to be a structure formed by Y1767 and F1760 (Nau et al., 2000). Although there is a long distance between these two binding sites on the primary sequence, the spatial proximity may exist to provide the necessary conditions for amitriptyline to impair the interaction between Nav1.5 and ankyrin-G. On the other hand, we discovered that the long-term effect of amitriptyline could destroy the Nav1.5-dystrophin interaction. It should be noted that dystrophin was functionally related to the cardiac sodium channels located on the lateral membrane and was responsible for the sodium current originating from those channels (Gavillet et al., 2006). Although we could not distinguish intercalated disks from the lateral membrane on neonatal rat ventricular myocytes, these results did suggest that the Nav1.5 channels located on both intercalated disks and the lateral membrane might be involved in the mechanisms of amitriptyline-induced BrS.

In line with the findings on amitriptyline, we found that clomipramine also showed a stronger blockade effect on Nav1.5 by long-term application than by acute application. Inconsistently, nortriptyline showed similar blockade effects on Nav1.5 with long-term or acute application, whereas desipramine failed to block Nav1.5 by either long-term or acute application. Although different antidepressants may have different capacities in the blockade of Nav1.5, the long-term effect of drugs should be considered seriously in further studies referring to drug-induced BrS.

We acknowledge the limitations in the present study. The adult rat myocytes should be a better choice to observe the specific location of Nav1.5 on intercalated disks and the lateral membrane. However, considering the difficulty regarding the long-term culture of adult rat myocytes, we used neonatal rat myocytes instead, without observation of the intercalated disk and lateral membrane. In addition, the impacts of other antidepressants (including clomipramine, nortriptyline, and desipramine) on the expression and distribution of the Nav1.5 protein were not studied in the present study but deserve further research. More importantly, evidence is accumulating that Nav1.5 channels were part of the sodium channel macromolecular complexes and trafficking of Nav1.5 proteins were regulated by many Nav1.5-interacting proteins, such as SAP97, MOG1, Plakophilin-2, beta subunits (Shao et al., 2009; Abriel et al., 2015). In the present study, we only chose two typical Nav1.5 proteins for experiments. Ankyrin-G mainly regulates the Nav1.5 proteins on intercalated disk membrane and dystrophin regulates the Nav1.5 proteins on lateral membranes, respectively. However, the function of Nav1.5 depends on the complicated cooperation among multiple Nav1.5-interacting proteins. Apart from above Nav1.5-interacting proteins, the Kir2.x channels have also been proved to function within the macromolecular complexes. Nav1.5/Kir2.x reciprocal modulation has been elucidated previously through a critical domain of Nav1.5, which increases the sodium and potassium currents (Matamoros et al., 2016). If the critical domain could be influenced by amitriptyline, currents of both Nav1.5 and Kir2.x would decrease, which could give rise to Brs. Meanwhile, Nav1.5 and Kir2.x interact with common partners including ankyrin-G and dystrophin (Willis et al., 2015). Thus Kir2.x and other interacting proteins may as well paly important roles in antidepressant-induced BrS, which could be further explored in the future. Still, the present findings demonstrated for the first time that the long-term effect should be considered important in BrS induced by amitriptyline. Impaired Nav1.5 trafficking, regulated by Nav1.5-ankyrin-G and Nav1.5-dystrophin interaction, was suggested to be the mechanism related to such a long-term effect. The present study may provide a new clue for the further investigation on BrS and experimental basis for rational clinical drug use in the future.

## Author Contributions

XC carried out experiments and wrote the manuscript. CZ and HZ assisted with the Western blot experiments. YZ and ZC assisted with data analysis. HW and XR assisted with path clamp experiments. LG assisted to write the manuscript. JZ assisted to design experiments. YL designed experiments.

## Conflict of Interest Statement

The authors declare that the research was conducted in the absence of any commercial or financial relationships that could be construed as a potential conflict of interest. The reviewer RV and handling Editor declared their shared affiliation at the time of the review.

## Footnotes

1 www.brugadadrugs.org

## References

[B1] Abriel H. Rougier J. S. Jalife J. (2015). Ion channel macromolecular complexes in cardiomyocytes: roles in sudden cardiac death. *Circ. Res.* 116 1971–1988. 10.1161/CIRCRESAHA.116.305017 26044251 PMC4471480

[B2] Bardai A. Amin A. S. Blom M. T. Bezzina C. R. Berdowski J. Langendijk P. N. (2013). Sudden cardiac arrest associated with use of a non-cardiac drug that reduces cardiac excitability: evidence from bench, bedside and community. *Eur. Heart J.* 34 1506–1516. 10.1093/eurheartj/eht054 23425522

[B3] Casini S. Tan H. L. Demirayak I. Remme C. A. Amin A. S. Scicluna B. P. (2010). Tubulin polymerization modifies cardiac sodium channel expression and gating. *Cardiovasc. Res.* 85 691–700. 10.1093/cvr/cvp352 19861310

[B4] Chen X. Zhang Y. Xu B. Cai Z. Wang L. Tian J. (2016). The mitochondrial calcium uniporter is involved in mitochondrial calcium cycle dysfunction: underlying mechanism of hypertension associated with mitochondrial tRNA(Ile) A4263G mutation. *Int. J. Biochem. Cell Biol.* 78 307–314. 10.1016/j.biocel.2016.07.018 27471128

[B5] Chow B. J. Gollob M. Birnie D. (2005). Brugada syndrome precipitated by a tricyclic antidepressant. *Heart* 91:651. 10.1136/hrt.2004.049593 15831654 PMC1768892

[B6] Donovan B. T. Bakshi T. Galbraith S. E. Nixon C. J. Payne L. A. Martens S. F. (2011). Utility of frozen cell lines in medium throughput electrophysiology screening of hERG and NaV1.5 blockade. *J. Pharmacol. Toxicol. Methods* 64 269–276. 10.1016/j.vascn.2011.09.002 21996251

[B7] Garrido J. J. Giraud P. Carlier E. Fernandes F. Moussif A. Fache M. P. (2003). Targeting motif involved in sodium channel clustering at the axonal initial segment. *Science* 300 2091–2094. 10.1126/science.1085167 12829783

[B8] Gavillet B. Rougier J. S. Domenighetti A. A. Behar R. Boixel C. Ruchat P. (2006). Cardiac sodium channel Nav1.5 is regulated by a multiprotein complex composed of syntrophins and dystrophin. *Circ. Res.* 99 407–414. 10.1161/01.RES.0000237466.13252.5e 16857961

[B9] Goldgran-Toledano D. Sideris G. Kevorkian J. P. (2002). Overdose of cyclic antidepressants and the Brugada syndrome. *N. Engl. J. Med.* 346 1591–1592. 10.1056/NEJM200205163462020 12015405

[B10] Herfst L. J. Rook M. B. Jongsma H. J. (2004). Trafficking and functional expression of cardiac Na+ channels. *J. Mol. Cell. Cardiol.* 36 185–193. 10.1016/j.yjmcc.2003.11.014 14871545

[B11] Juang J. M. Chen C. Y. Chen Y. H. Wu I. C. Hsu C. C. Chen L. N. (2015). Prevalence and prognosis of Brugada electrocardiogram patterns in an elderly han chinese population: a nation-wide community-based study (HALST cohort). *Europace* 17(Suppl. 2) ii54–ii62. 10.1093/europace/euv141 26842116

[B12] Kadota S. Pabon L. Reinecke H. Murry C. E. (2017). In vivo maturation of human induced pluripotent stem cell-derived cardiomyocytes in neonatal and adult rat hearts. *Stem Cell Rep.* 8 278–289. 10.1016/j.stemcr.2016.10.009 28065644 PMC5311430

[B13] Kofune M. Kofune T. Ohkubo K. Watanabe I. (2013). Electrocardiographic changes upon tricyclic antidepressant administration mimicking Brugada syndrome. *Intern. Med.* 52:1427. 10.2169/internalmedicine.52.0016 23774564

[B14] Lemaillet G. Walker B. Lambert S. (2003). Identification of a conserved ankyrin-binding motif in the family of sodium channel alpha subunits. *J. Biol. Chem.* 278 27333–27339. 10.1074/jbc.M303327200 12716895

[B15] Liang J. Liu X. Pan M. Dai W. Dong Z. Wang X. (2014). Blockade of Nav1.8 currents in nociceptive trigeminal neurons contributes to anti-trigeminovascular nociceptive effect of amitriptyline. *Neuromolecular Med.* 16 308–321. 10.1007/s12017-013-8282-6 24292897

[B16] Lowe J. S. Palygin O. Bhasin N. Hund T. J. Boyden P. A. Shibata E. (2008). Voltage-gated Nav channel targeting in the heart requires an ankyrin-G dependent cellular pathway. *J. Cell. Biol.* 180 173–186. 10.1083/jcb.200710107 18180363 PMC2213608

[B17] Makara M. A. Curran J. Little S. C. Musa H. Polina I. Smith S. A. (2014). Ankyrin-G coordinates intercalated disc signaling platform to regulate cardiac excitability in vivo. *Circ. Res.* 115 929–938. 10.1161/CIRCRESAHA.115.305154 25239140 PMC4224970

[B18] Matamoros M. Pérez-Hernández M. Guerrero-Serna G. Amorós I. Barana A. Núñez M. (2016). Nav1.5 N-terminal domain binding to α1-syntrophin increases membrane density of human Kir2.1, Kir2.2 and Nav1.5 channels. *Cardiovasc. Res.* 110 279–290. 10.1093/cvr/cvw009 26786162 PMC4836625

[B19] Minoura Y. Di Diego J. M. Barajas-Martinez H. Zygmunt A. C. Hu D. Sicouri S. (2012). Ionic and cellular mechanisms underlying the development of acquired Brugada syndrome in patients treated with antidepressants. *J. Cardiovasc. Electrophysiol.* 23 423–432. 10.1111/j.1540-8167.2011.02196.x 22034916 PMC3468832

[B20] Mohler P. J. Rivolta I. Napolitano C. LeMaillet G. Lambert S. Priori S. G. (2004). Nav1.5 E1053K mutation causing Brugada syndrome blocks binding to ankyrin-G and expression of Nav1.5 on the surface of cardiomyocytes. *Proc. Natl. Acad. Sci. U.S.A.* 101 17533–17538. 10.1073/pnas.0403711101 15579534 PMC536011

[B21] Murakoshi N. Aonuma K. (2013). Epidemiology of arrhythmias and sudden cardiac death in Asia. *Circ. J.* 77 2419–2431. 10.1253/circj.CJ-13-1129 24067274

[B22] Nau C. Seaver M. Wang S. Y. Wang G. K. (2000). Block of human heart hH1 sodium channels by amitriptyline. *J. Pharmacol. Exp. Ther.* 292 1015–1023. 10688618

[B23] Ng C. T. Ong H. Y. Cheok C. Chua T. S. Ching C. K. (2012). Prevalence of electrocardiographic abnormalities in an unselected young male multi-ethnic South-East Asian population undergoing pre-participation cardiovascular screening: results of the Singapore armed forces electrocardiogram and echocardiogram screening protocol. *Europace* 14 1018–1024. 10.1093/europace/eur424 22308089

[B24] Pancrazio J. J. Kamatchi G. L. Roscoe A. K. Lynch C. III (1998). Inhibition of neuronal Na+ channels by antidepressant drugs. *J. Pharmacol. Exp. Ther.* 284 208–214. 9435180

[B25] Priori S. G. Wilde A. A. Horie M. Cho Y. Behr E. R. Berul C. (2013). HRS/EHRA/APHRS expert consensus statement on the diagnosis and management of patients with inherited primary arrhythmia syndromes: document endorsed by HRS, EHRA and APHRS in May 2013 and by ACCF, AHA, PACES and AEPC in June 2013. *Heart Rhythm* 10 1932–1963. 10.1016/j.hrthm.2013.05.014 24011539

[B26] Ray W. A. Meredith S. Thapa P. B. Hall K. Murray K. T. (2004). Cyclic antidepressants and the risk of sudden cardiac death. *Clin. Pharmacol. Ther.* 75 234–241. 10.1016/j.clpt.2003.09.019 15001975

[B27] Shao D. Okuse K. Djamgoz M. B. (2009). Protein-protein interactions involving voltage-gated sodium channels: post-translational regulation, intracellular trafficking and functional expression. *Int. J. Biochem. Cell Biol.* 41 1471–1481. 10.1016/j.biocel.2009.01.016 19401147

[B28] Staudacher I. Wang L. Wan X. Obers S. Wenzel W. Tristram F. (2011). HERG K^+^ channel-associated cardiac effects of the antidepressant drug desipramine. *Naunyn Schmiedebergs Arch. Pharmacol.* 383 119–139. 10.1007/s00210-010-0583-9 21120454

[B29] Wang G. K. Russell C. Wang S. Y. (2004). State-dependent block of voltage-gated Na+ channels by amitriptyline via the local anesthetic receptor and its implication for neuropathic pain. *Pain* 110 166–174. 10.1016/j.pain.2004.03.018 15275764

[B30] Willis B. C. Ponce-Balbuena D. Jalife J. (2015). Protein assemblies of sodium and inward rectifier potassium channels control cardiac excitability and arrhythmogenesis. *Am. J. Physiol. Heart Circ. Physiol.* 308 H1463–H1473. 10.1152/ajpheart.00176.2015 25862830 PMC4469872

[B31] Wu L. Li H. Li X. Chen Y. Zhang Q. Cheng Z. (2017). Peptidomic analysis of cultured cardiomyocytes exposed to acute ischemic-hypoxia. *Cell. Physiol. Biochem.* 41 358–368. 10.1159/000456282 28135715

[B32] Yang T. Chun Y. W. Stroud D. M. Mosley J. D. Knollmann B. C. Hong C. (2014). Screening for acute IKr block is insufficient to detect torsades de pointes liability: role of late sodium current. *Circulation* 130 224–234. 10.1161/CIRCULATIONAHA.113.007765 24895457 PMC4101031

[B33] Zhang C. Chen B. Guo A. Zhu Y. Miller J. D. Gao S. (2014). Microtubule-mediated defects in junctophilin-2 trafficking contribute to myocyte transverse-tubule remodeling and Ca^2+^ handling dysfunction in heart failure. *Circulation* 129 1742–1750. 10.1161/CIRCULATIONAHA.113.008452 24519927 PMC4006305

[B34] Zhang J. Chen Y. Yang J. Xu B. Wen Y. Xiang G. (2015). Electrophysiological and trafficking defects of the SCN5A T353I mutation in Brugada syndrome are rescued by alpha-allocryptopine. *Eur. J. Pharmacol.* 746 333–343. 10.1016/j.ejphar.2014.09.028 25261036

